# *N*-Docosahexaenoylethanolamine ameliorates LPS-induced neuroinflammation via cAMP/PKA-dependent signaling

**DOI:** 10.1186/s12974-016-0751-z

**Published:** 2016-11-04

**Authors:** Taeyeop Park, Huazhen Chen, Karl Kevala, Ji-Won Lee, Hee-Yong Kim

**Affiliations:** 1 Laboratory of Molecular Signaling, National Institute on Alcohol Abuse and Alcoholism, National Institutes of Health, 5625 Fishers Lane, Rockville, MD 20852 USA; 2 National Institute of Alcohol Abuse and Alcoholism, National Institutes of Health, 5625 Fishers Lane, Rm. 3N-07, Bethesda, MD 20892-9410 USA

**Keywords:** Synaptamide, Microglia, NF-κB, Docosahexaenoic acid, Cytokine, iNOS, CCL2, Iba-1, GFAP, FAAH

## Abstract

**Background:**

Brain inflammation has been implicated as a critical mechanism responsible for the progression of neurodegeneration and characterized by glial cell activation accompanied by production of inflammation-related cytokines and chemokines. Growing evidence also suggests that metabolites derived from docosahexaenoic acid (DHA) have anti-inflammatory and pro-resolving effects; however, the possible role of *N*-docosahexaenoylethanolamine (synaptamide), an endogenous neurogenic and synaptogenic metabolite of DHA, in inflammation, is largely unknown. (The term “synaptamide” instead of “DHEA” was used for *N*-docosahexaenoylethanolamine since DHEA is a widely used and accepted term for the steroid, dehydroepiandrosterone.) In the present study, we tested this possibility using a lipopolysaccharide (LPS)-induced neuroinflammation model both in vitro and in vivo.

**Methods:**

For in vitro studies, we used P3 primary rat microglia and immortalized murine microglia cells (BV2) to assess synaptamide effects on LPS-induced cytokine/chemokine/iNOS (inducible nitric oxide synthase) expression by quantitative PCR (qPCR) and enzyme-linked immunosorbent assay (ELISA). To evaluate in vivo effects, mice were intraperitoneally (i.p.) injected with LPS followed by synaptamide, and expression of proinflammatory mediators was measured by qPCR and western blot analysis. Activation of microglia and astrocyte in the brain was examined by Iba-1 and GFAP immunostaining.

**Results:**

Synaptamide significantly reduced LPS-induced production of TNF-α and NO in cultured microglia cells. Synaptamide increased intracellular cAMP levels, phosphorylation of PKA, and phosphorylation of CREB but suppressed LPS-induced nuclear translocation of NF-κB p65. Conversely, adenylyl cyclase or PKA inhibitors abolished the synaptamide effect on p65 translocation as well as TNF-α and iNOS expression. Administration of synaptamide following LPS injection (i.p.) significantly reduced neuroinflammatory responses, such as microglia activation and mRNA expression of inflammatory cytokines, chemokine, and iNOS in the brain.

**Conclusions:**

DHA-derived synaptamide is a potent suppressor of neuroinflammation in an LPS-induced model, by enhancing cAMP/PKA signaling and inhibiting NF-κB activation. The anti-inflammatory capability of synaptamide may provide a new therapeutic avenue to ameliorate the inflammation-associated neurodegenerative conditions.

**Electronic supplementary material:**

The online version of this article (doi:10.1186/s12974-016-0751-z) contains supplementary material, which is available to authorized users.

## Background

Inflammation is a widely accepted common denominator among various neuropathological processes and has been implicated as a critical mechanism responsible for the progression of neurodegenerative disorders including Parkinson’s disease [1], Alzheimer’s disease [2], and multiple sclerosis [3] as well as traumatic brain injury [4]. Microglia are resident immune-like glial cells in the central nervous system that contribute to neuroinflammation. Activated microglia secrete proinflammatory cytokines, chemokines, and oxygen radicals [5], often leading to the development of cerebral damage under various pathologic conditions. Therefore, microglia activation has been regarded as a strategic target for therapeutic intervention in brain diseases associated with inflammation.

Docosahexaenoic acid (22:6n-3, DHA), the major polyunsaturated fatty acid highly enriched in the brain, is known to exert neurotrophic and neuroprotective functions [6–8]. DHA was shown to suppress inflammation in various cells including microglia cells [9–11], suggesting neuroinflammation is one of the multiple target mechanisms for its neuroprotective action. Recently, we have demonstrated that *N*-docosahexaenoylethanolamine (synaptamide), an endogenous metabolite of DHA, potently induces neurogenic differentiation of neural stem cells (NSC) and promotes neurite growth and synaptogenesis in developing neurons [12, 13]. The synaptamide effect on neurogenic differentiation was shown to be mediated through the cAMP/PKA signaling pathway [12, 14] which is also an inhibitory target for lipopolysaccharide (LPS)-induced NF-κB activation leading to inflammation [15]. Inhibitory effects of this DHA metabolite on inflammation has indeed been reported [16–18] and were shown to be greater compared to its parent compound DHA [17]. Nevertheless, the potential role of synaptamide in neuroinflammation has not been explored. In this study, we investigated the effects of synaptamide on LPS-induced neuroinflammation in both in vitro and in vivo models, along with underlying signaling mechanisms. We found that synaptamide potently inhibits LPS-induced proinflammatory cytokine/chemokine production and nuclear translocation of NF-κB subunit p65 in microglia cells through upregulating cAMP/PKA signaling. In vivo administration of synaptamide significantly suppressed microglia activation induced by the LPS injection (i.p.) and almost completely abolished cytokine production in mouse brains. The anti-neuroinflammatory effects of synaptamide suggest a potential new therapeutic avenue for inflammation-related neurodegenerative conditions or brain injury.

## Methods

### Chemicals and antibodies

Dulbecco’s Modified Eagle Medium/Ham’a F12 (DMEM/F12 1:1) and bovine calf serum were purchased from Life Technologies Corporation (Carlsbad, CA, USA). Ketoconazole, Indomethacin, SQ 22536, H-89, lipopolysaccharides from *Escherichia coli* 055:B5 and forskolin were purchased from Sigma-Aldrich (St. Louis, MO, USA), and normal goat serum was acquired from Dako (Carpentaria, CA, USA). Anti-Iba-1 antibody and anti-glial fibrillary acidic protein (GFAP) antibody were purchased from Wako (Richmond, VA, USA) and Sigma-Aldrich (St. Louise, MO, USA), respectively. Antibodies to PKA, CREB, and GAPDH were purchased from Cell Signaling. Heneicosanoic acid was from Nu-Chek Prep (Elysian, MN). Butylated hydroxytoluene (BHT), LY311727, neomycin sulfate, and 10% boron trifluoride-methanol (BF_3_/methanol) were from Sigma-Aldrich (St. Louis, MO). Methyl arachidonyl fluorophosphonate (MAFP), bromoenol lactone (BEL), and ZLJ-6 were obtained from Cayman Chemical (Ann Arbor, MI). 2-TEDC was from Tocris Bioscience (Bristol, UK). HPLC grade water and acetonitrile were from Avantor Performance Materials (Center Valley, PA). Other solvents, BCA protein assay kit, and acetic acid were from Fisher Scientific (Pittsburgh, PA)

### Cell culture

Primary microglia cultures were prepared from 3–4-day-old Wistar rats (Charles River Laboratories) according to the procedure previously described [19]. Briefly, after removing the meninges and blood vessels, the forebrain was dissociated by repeated pipetting in DMEM/F12 (Life Technologies) containing 10 % bovine calf serum (BCS) and gentamicin and then filtered through a 70-μm cell strainer. Cells were collected by centrifugation, resuspended in 10 % BCS DMEM/F12, and cultured in 75-cm^2^ flasks (Nunc, Roskilde, Denmark) in a humidified atmosphere of 5 % CO^2^/95 % air at 37 °C. After 10–14 days, microglia were isolated by shaking the mixed glia-containing flasks. The floating cells were seeded onto plastic tissue culture dishes and incubated at 37 °C. The attached cells were harvested and seeded onto plates for further experiments. The purity of microglia greater than 90 % was attained consistently (Additional file 1: Figure S1). BV2 cells, a mouse microglial cell line that was a kind gift from Dr. Mason (NIEHS, NIH), were cultured in Dulbecco’s modified Eagle’s medium (DMEM) (ATCC) containing 10 % fetal bovine serum (GIBCO) with 1 % penicillin/streptomycin (Invitrogen). Microglia or BV2 cells were treated with LPS (Sigma-Aldrich) at a concentration of 100 ng/mL or synaptamide at indicated concentrations.

### Animals

C57BL/6J mice were purchased from Charles River Laboratories (Portage, MI, USA), and FAAH KO mice were kind gift from Dr. Cravatt’s laboratory. All experiments in this study were carried out in accordance with the guiding principles for the care and use of animals approved by the National Institute on Alcohol Abuse and Alcoholism (LMS-HK-13, LMS-HK-41). Mice 8–12 weeks of age received intraperitoneal (i.p.) injections of saline or 1.0 mg/kg LPS (*E. coli*, serotype 055:B5, Sigma) followed by synaptamide (2 or 5 mg/kg). At 2 h after final injection, mice were deeply anesthetized with isoflurane and perfused quickly with chilled phosphate-buffer saline (PBS) for RNA isolation or chilled PBS containing 4 % paraformaldehyde for immunostaining.

### siRNA transfection

BV2 cells maintained in DMEM containing 10 % FBS with 1 % penicillin/streptomycin were transiently transfected with SignalSilence PKA C-α small interfering ribonucleic acid (siRNA) or scrambled control siRNA (Cell Signaling Technology) at the final concentration of 100 nM using Lipofectamine 2000 (Invitrogen) with Opti-MEM according to the manufacturer’s protocol.

### Immunostaining

BV2 murine microglia cells stimulated with LPS (100 ng/mL) were fixed with 4 % paraformaldehyde for 30 min at room temperature, washed with 0.1 M Tris-buffered solution (pH 7.5, TBS), blocked with 10 % normal goat serum in TBS containing 0.3 % Triton X-100 at room temperature for 60 min, and incubated with mouse-anti-p65 antibody (1:400) at 4 °C overnight. The cells were washed with TBS and incubated with Alexa Fluor 488-conjugated secondary antibodies (1:1000, Life Technologies Corporation) at room temperature for 60 min. To visualize nuclei, cells were counterstained with 2 μg/mL 4′,6-diamidino-2-phenylindole (DAPI). Finally, the cells were mounted with 80 % (vol/vol) glycerol, visualized under a fluorescent microscope (IX81, Olympus Corp., Tokyo, Japan), and the image data were processed using MetaMorph (Molecular Devices, Sunnyvale, CA, USA) for quantitative information. For staining brain samples, mice were anesthetized with isoflurane and transcardially perfused with 0.1 M phosphate buffer (PB, pH 7.4) containing 4 % paraformaldehyde (wt/vol), and the brains were carefully removed. After overnight fixing in 4 % paraformaldehyde solution followed by submerging in 30 % sucrose solution at 4 °C, the brains were embedded with O.C.T. compound (Tissue-Tek, 4583) medium, frozen on dry ice, and stored at −80 °C freezer until use. Coronal sections (30 μm) were prepared using Leica Cryostat and stored into the cryoprotective solution at −20 °C. Brain sections at the approximately same position from Bregma were immunostained using anti-Iba-1 (Wako, catalogue number 019-19741) or anti-GFAP (Sigma, catalogue number G9269) antibody followed by Alexa fluor-488-conjugated F (ab’) 2 fragment goat anti-rabbit IgG (Jackson ImmunoResearch labs, catalogue number 111-546-003).

### Cell fractionation

Cells were harvested at 4 °C and lysed, and cytoplasmic and nuclear extracts were prepared using an NE-PER nuclear and cytoplasmic extraction reagent kit (Pierce Biotechnology) as described by the manufacturer. Protease inhibitors were added (complete mini-protease inhibitor cocktail tablets, obtained from Roche Diagnostic, Meylan, France). Briefly, 10 mg of cells was resuspended in 50 μL cytoplasmic extraction reagent I (CERI), mixed, and incubated on ice for 10 min; 2.75 μL of cytoplasmic extraction reagent II (CERII) was then added, followed by mixing and incubation on ice for 1 min. The intact nuclei were pelleted, and the supernatant cytoplasmic extract was collected. The nuclei were resuspended in 25 μL of ice-cold nuclear extraction reagent (NER), incubated on ice repeatedly, and centrifuged to obtain the supernatant containing nuclear proteins. Protein concentrations were determined using the micro-bicinchoninic acid (BCA) protein assay kit from Pierce Biotechnology.

### LC/MS for synaptamide measurement

Samples were frozen at −80 °C until analysis. C57 mouse brains, not including cerebellum or olfactory bulbs, were homogenized on wet ice using a motorized Teflon pestle homogenizer in 1–2 mL 50 %/50 % methanol/water containing 25 μg/mL β-hydroxytoluene (BHT) as an antioxidant and the following phospholipase, cyclooxygenase, lipoxygenase, or fatty acid amide hydrolase inhibitors: 10 μM BEL, 30 μM MAFP, 50 μM ZLJ-6, 25 μM 2-TEDC, 500 μM neomycin sulfate, or 200 μM LY311727. The protein content of the homogenate was measured using BCA protein assay kit. Synaptamide, d4-synaptamide, and anandamide were determined as follows. In brief, 300 μL aliquots of homogenate were brought to 70 % MeOH and spun in a microcentrifuge at 18,000 RCF at 4 °C for 20 min in the presence of either d4-synaptamide or d4-anandamide as an internal standard. Supernatants were loaded onto 30 mg Strata-X 33 μm polymeric reverse phase SPE cartridges (Phenomenex, Torrance, CA) that had been prewashed with 2 mL methanol followed by 2 mL water. Columns were then washed with 10 mL water and N-acyl ethanolamides eluted with 1.25 mL methanol containing 50 μg/mL BHT. The eluant was dried under nitrogen and suspended in a small volume of methanol. Five microliters of the extract was analyzed by reverse phase HPLC-MS-MS using a Zorbax Eclipse Plus C18 column (2.1 × 50 mm, 1.8 μm) (Agilent Technologies, Santa Clara, CA) coupled to a Q-Exactive mass spectrometer (Thermo Scientific, Waltham, MA) operating in the positive ion targeted MS-MS mode. HPLC solvents contained 0.01 % acetic acid, and the ternary gradient consisted of two parts: first, a change from acetonitrile/methanol/water 16.3/30/53.7 to 48.7/15/36.3 in 5 min, followed by a linear gradient to 18.1/68.4/13.5 in 22 min. *N*-Acyl ethanolamides were quantitated against the corresponding internal standard using the ethanolamine fragment.

### Western blot analysis

Proteins from cell lysates (50 μg protein) were separated via SDS–PAGE and electroblotted onto a polyvinylidene difluoride (PVDF) membrane for 90 min at 100 V at 4 °C. After blocking with 5 % BSA for 60 min in TBS-T (20 mM Tris–HCl, pH 7.5, 50 mM NaCl, 0.1 % Tween 20), the membrane was incubated overnight with primary antibodies in 5 % BSA at 4 °C, washed with TBS-T buffer, and incubated for 60 min in anti-rabbit or anti-mouse IgG–horseradish peroxidase (Santa Cruz Biotechnology, Santa Cruz, CA, USA) secondary antibodies diluted in 5 % BSA (1:3000). After treating with chemiluminescence reagents (Thermo Fisher Scientific, Rockford, IL, USA), protein bands were detected and quantified using a Kodak Gel Logic 440 imaging system with ImageJ software. Unless specified otherwise, glyceraldehyde 3-phosphate dehydrogenase (GAPDH) was used as the loading control for all western blotting analyses.

### cAMP assay

Cultured microglia (2.5 × 10^5^ cells in 0.5 mL) were treated with synaptamide for 15 min, and the cAMP level was determined using cyclicAMP XP® assay kit (Cell Signaling, Danvers, MA) according to the manufacturer’s protocol. Briefly, cells were lysed using a lysis buffer including protease inhibitor cocktail (Cell Signaling), and the cell lysate was added to the cyclicAMP XP® assay kit to displace HRP-linked cAMP bound to an anti-cAMP XP® Rabbit mAb immobilized onto a 96-well plate. After removing displaced HRP-linked cAMP, HRP substrate TMB was added and cAMP concentration was measured colorimetrically at 450 nm.

### RNA isolation and quantitative RT-PCR

Total RNA was extracted using Trizol according to manufacturer’s protocol (Invitrogen, UK). RNA was treated with DNase I to remove any contaminating genomic DNA (RQ1 RNase-free DNase; Promega). RNA was then used for cDNA synthesis applying reverse transcription reagents (Applied Biosystems Inc., Foster City, CA, USA). Expression of messenger RNA (mRNA) for TNF-α, iNOS, IL-1β, IL-6, IL-10, CCL2, and GAPDH were measured via a SYBR green-based real-time RT-PCR assay. Samples were analyzed in triplicate on an ABI Prism 7900HT sequence detection system and QuantiTect SYBR Green PCR kit (Qiagen, CA, USA). The amplification conditions were 50 °C for 2 min then 95 °C for 15 min, followed by 40 cycles of 95 °C for 15 s and 55 °C for 30 s. SDS 2.4 software (Applied Biosystems Inc., Foster City, CA, USA) was used to calculate the cycle threshold (Ct) in real-time assays. ΔΔCt was used to estimate the differential gene expression between samples (Method 2001 25:402-408). The relative expression of mRNA was calculated after normalization to GAPDH mRNA. Primer sequences are indicated below as a Table.

**Table:** 

		For mice	For rat
TNF-α	Forward	CCCTCCAGAAAAGACACCATG	CAGATGGGCTGTACCTTATC
	Reverse	GCCACAAGCAGGAATGAGAAG	GGTATGAAATGGCAAATCGG
IL-1β	Forward	CCACCTTTTGACAGTGATGA	CTAGTGTGTGATGTTCCCAT
	Reverse	GAGATTTGAAGCTGGATGCT	CCACTTGTTGGCTTATGTTC
IL-6	Forward	GTCGGAGGCTTAATTACACA	CTTCCCTACTTCACAAGTCC
	Reverse	TTTTCTGCAAGTGCATCATC	TTCCAAGATCTCCCTGAGAA
IL-10	Forward	AGCCTTATCGGAAATGATCC	TCTACAAGGCCATGAATGAG
	Reverse	GGGAATTCAAATGCTCCTTG	CGGGTGGTTCAATTTTTCAT
CCL2	Forward	GGATCGGAACCAAATGAGAT	CTACTCATTCACTGGCAAGA
	Reverse	ATTTACGGGTCAACTTCACA	TCTTGAGCTTGGTGACAAAT
iNOS	Forward	CCTAGTCAACTGCAAGAGAA	TGGGTCTTGTTAGCCTAGT
	Reverse	TTTCAGGTCACTTTGGTAGG	TCACCTTGGTAGGATTTGAC
GAPDH	Forward	CCACTCACGGCAAATTCAAC	AGGATACTGAGAGCAAGAGA
	Reverse	CTCCACGACATACTCAGCAC	TTGATGGTATTCGAGAGAAGG

### Nitrate/nitrite assays

The amount of NO produced by cells was indirectly measured by the formation of nitrate (NO_3_^−^) and nitrite (NO_2_^−^) (two stable end products of NO) in culture supernatants. The total amounts of nitrate and nitrite were determined with a nitrate/nitrite assay kit (Cayman Chemical Co., Ann Arbor, MI), using the manufacturer’s protocols. Briefly, supernatant (100 μL) was incubated with nitrate reductase (20 μL) at room temperature for 1 h, followed by incubation with 100 μL Griess reagent (1 % sulfanilic acid, 0.1 % naphthyl ethylenediamine dihydrochloride, 2.5 % phosphoric acid) for 10 min. The optical density of each sample was analyzed at 540 nm in a microplate reader with sodium nitrite as a standard.

### ELISA assays

Following the stimulation of the cells with LPS and/or synaptamide, supernatants were collected and assayed using sandwich ELISA with their well-matched antibodies. TNF-α was detected using ELISA kits (Invitrogen Life Technologies, Frederick, MD, USA) according to the manufacturer’s instructions [20].

### Statistical analysis

The results are expressed as means ± SEM for triplicate samples and represent at least three independent experiments. Statistical analyses were conducted using one-way ANOVA followed by Tukey’s post hoc test (Prism software, GraphPad Inc., CA, USA). The statistical significance was determined at *p* < 0.05. Means designated with the same letter are not significantly different.

## Results

### Synaptamide inhibits LPS-induced TNF-α/iNOS expression in microglia cells

**Fig. 1:**
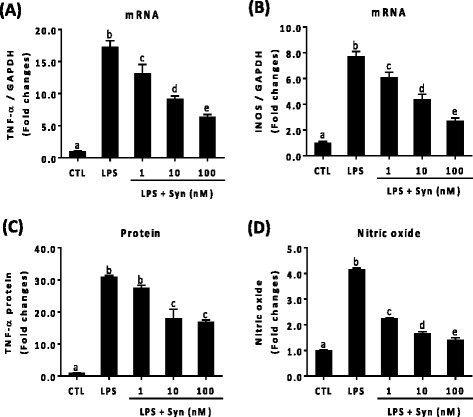
Inhibitory effect of synaptamide on LPS-induced expression of TNF-α and iNOS in microglia cells. Primary rat microglia cells were incubated with LPS (100 ng/mL) and/or synaptamide at the indicated concentration. Synaptamide was added immediately after LPS exposure. Levels of TNF-α (**a**) and iNOS (**b**) mRNA were determined by qPCR after 1 h incubation. After 24 h, accumulated levels of TNF-α (**c**) and nitric oxide (**d**) in the media were measured by ELISA and Griess reaction. The data represent the mean ± SEM at least three independent experiments. Means designated with the same letter are not significantly different. One-way ANOVA followed by Tukey’s post hoc test (Prism Software, SigmaStat)

To characterize the synaptamide effects on neuroinflammation, the LPS-stimulated expression of TNF-α and iNOS (inducible nitric oxide synthases) were measured as representative proinflammatory cytokine and nitrogen species, respectively, at the mRNA and protein/nitrogen species product levels (Fig. 1), using primary cultures of rat microglia. After optimization of the LPS dose (10–1000 ng/mL) and time course (30 min–4 h) for the induction of proinflammatory cytokine expression (data not shown), the TNF-α/iNOS expression was evaluated after stimulation with LPS at 100 ng/mL for 1 h. Primary microglia cells significantly increased the expression of TNF-α (17.34 ± 1.83-fold) and iNOS (7.74 ± 0.73-fold) mRNA relative to the control level. Synaptamide dose-dependently suppressed LPS-induced TNF-α and iNOS mRNA expression. After treatment with 1, 10, and 100 nM synaptamide, LPS-induced TNF-α mRNA expression decreased from 17.34 ± 1.83 (fold increase) to 13.17 ± 1.95, 9.18 ± 1.34, and 6.37 ± 0.97, respectively (Fig. 1a). At these concentrations, synaptamide also decreased iNOS mRNA from 7.74 ± 0.73 (fold increase) to 6.09 ± 0.67, 4.37 ± 0.69, and 2.71 ± 0.39, respectively (Fig. 1b).

At 24 h after LPS treatment, marked increases in TNF-α protein (31.02 ± 0.52-fold) and nitric oxide (4.17 ± 0.05-fold) were observed in the rat microglial culture media, which was reduced by synaptamide dose-dependently (Fig. 1c, d). The BV2 murine microglia cells also produced similar results as synaptamide decreased LPS-induced increase in TNF-α and iNOS mRNA as well as TNF-α protein and nitric oxide (data not shown). The in vitro data obtained from these two microglia cultures indicated that synaptamide at nanomolar concentrations can significantly suppress the production of proinflammatory cytokine/nitrogen species induced under a LPS-stimulated condition.

### Synaptamide among fatty acyl ethanolamides uniquely suppresses LPS-induced TNF-α and iNOS expression

**Fig. 2:**
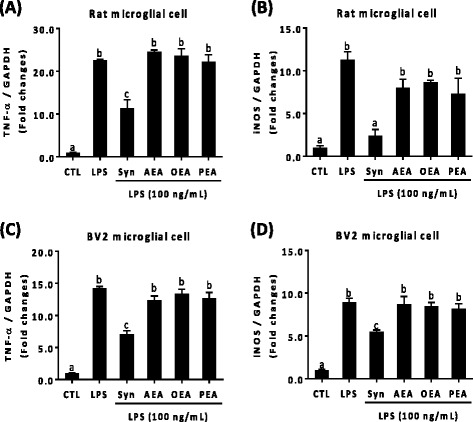
Suppression of LPS-induced TNF-α and iNOS expression specific to synaptamide. Cells were incubated with LPS (100 ng/mL) and acylethanolamides at 10 nM. Acylethanolamides were added immediately after LPS exposure. Levels of TNF-α and iNOS (**a**, **b**; rat primary microglial cell, **c**, **d**; BV2 murine microglial cell) mRNA were determined by qPCR after 1 h incubation. The data represent the mean ± SEM at least three independent experiments. Means designated with the same letter are not significantly different

We tested whether other fatty acyl ethanolamides such as arachidonoyl ethanolamine (AEA), oleoyl ethanolamide (OEA), and palmitoyl ethanolamine (PEA) exert similar anti-inflammatory activity. At 10 nM, only synaptamide significantly suppressed LPS-stimulated TNF-α and iNOS mRNA expression in primary microglia (Fig. 2a, b) and BV2 cells (Fig. 2c, d), suggesting that the observed inhibitory effect is specific to synaptamide. The LPS-induced expression of other proinflammatory cytokines such as IL-1β, IL-6, and CCL2 was similarly suppressed only by synaptamide in BV2 cells as shown in Additional file 1: Figure S4.

### Synaptamide decreases LPS-induced TNF-α expression through cAMP/PKA signaling pathway

**Fig. 3:**
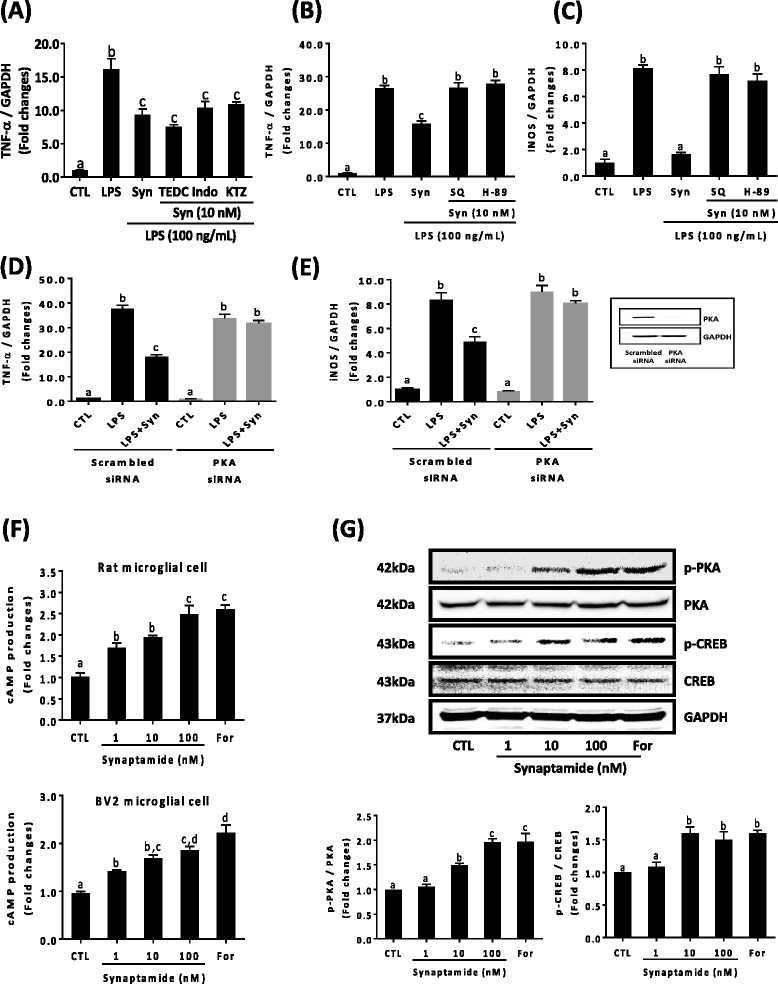
Involvement of cAMP/PKA signaling in the suppression of LPS-induced TNF-α expression by synaptamide. Microglia cells were incubated with LPS (100 ng/mL) and 10 nM synaptamide, and levels of TNF-α and iNOS mRNA were determined by qPCR at 1 h after incubation. 5-, 12-, 15-LOX inhibitor (2-TEDC, 10 μM); COX inhibitor (Indo, 10 μM); P450 inhibitor (KTZ, 10 μM) (**a**); adenylyl cyclase inhibitor (SQ 22536, 10 μM); and PKA inhibitor (H-89, 10 μM) (**b**, **c**) were added 30 min prior to LPS treatment. BV2 murine microglia cells were transfeted with PKA siRNA or scrambled control RNA for 48 h prior to the treatment with LPS (100 ng/mL) and synaptamide (10 nM) for 1 h (**d**, **e**). Successful knockdown of PKA expression is indicated in the *inset*. PKA primary rat microglia (**f**, *upper*) and BV2 (**f**, *bottom*) cells were treated with synaptamide (1–100 nM) or forskolin (For, 10 μM) as a positive control for 15 min, and cAMP levels were measured. BV2 cells were incubated with synaptamide at the indicated concentration for 30 min and subjected to immunoblot analysis using antibodies that specifically recognize phosphorylated and non-phosphorylated PKA, CREB (**g**). Graph values are mean ± SEM of protein levels of p-PKA/p-CREB relative to PKA/CREB, respectively (*n* = 3). Means designated with the same letter are not significantly different

Polyunsaturated fatty acids can be oxygenated to lipid mediators by cyclooxygenase (COX), lipoxygenase (LOX), and cytochrome P450 enzymes. Some of these metabolites are known to mediate short- and long-term inflammatory responses [21]. To investigate whether an oxygenated form of synaptamide is involved in the suppression of proinflammatory cytokine production, synaptamide effects were examined using specific oxygenase inhibitors (Fig. 3a). We found that 2-TEDC, indomethacin and ketoconazole (KTZ), specific inhibitors of LOX, COX, and cytochrome P450, respectively, did not affect the synaptamide-mediated inhibition of TNF-α expression in murine microglial cells, suggesting unlikely the involvement of an oxygenated metabolite. We have recently shown that the potent neurogenic activity of synaptamide is mediated via PKA/CREB activation [12]. Previous studies indicated cAMP/PKA/CREB signaling attenuates LPS-induced inflammatory responses by inhibiting NF-κB activation [22, 23]. Therefore, we tested whether synaptamide ameliorates the LPS-induced TNF-α and iNOS expression via cAMP/PKA pathway. Adenylyl cyclase inhibitor (SQ 22536, 10 μM) or PKA inhibitor (H-89, 10 μM) added to the culture media 30 min prior to LPS and synaptamide treatment completely blocked synaptamide-derived suppression of TNF-α and iNOS expression (Fig. 3b, c). To further confirm the role of PKA in synaptamide-mediated anti-inflammatory effect, cells were transfected with siRNA against PKA catalytic subunit α and scrambled control siRNA. Similar to the pharmacological inhibition, PKA knockdown by siRNA abolished the synaptamide-mediated expression of TNF-α and iNOS (Fig. 3d, e). Furthermore, synaptamide at 1–100 nM dose-dependently increased cellular cAMP production in both primary rat microglia (2.48 ± 0.37-fold increase at 100 nM, *p* < 0.032) (Fig. 3f, *upper panel*) and BV2 murine microglia cells (1.86 ± 0.17-fold increase at 100 nM, *p* < 0.001) (Fig. 3f, *bottom* *panel*). At the indicated concentrations, synaptamide also significantly increased phosphorylation of PKA and CREB in BV2 cells (Fig. 3g). These results indicate that synaptamide suppressed LPS-induced TNF-α and iNOS expression through the upregulation of cAMP and PKA signaling.

### Synaptamide inhibits LPS-induced p65 translocation

**Fig. 4:**
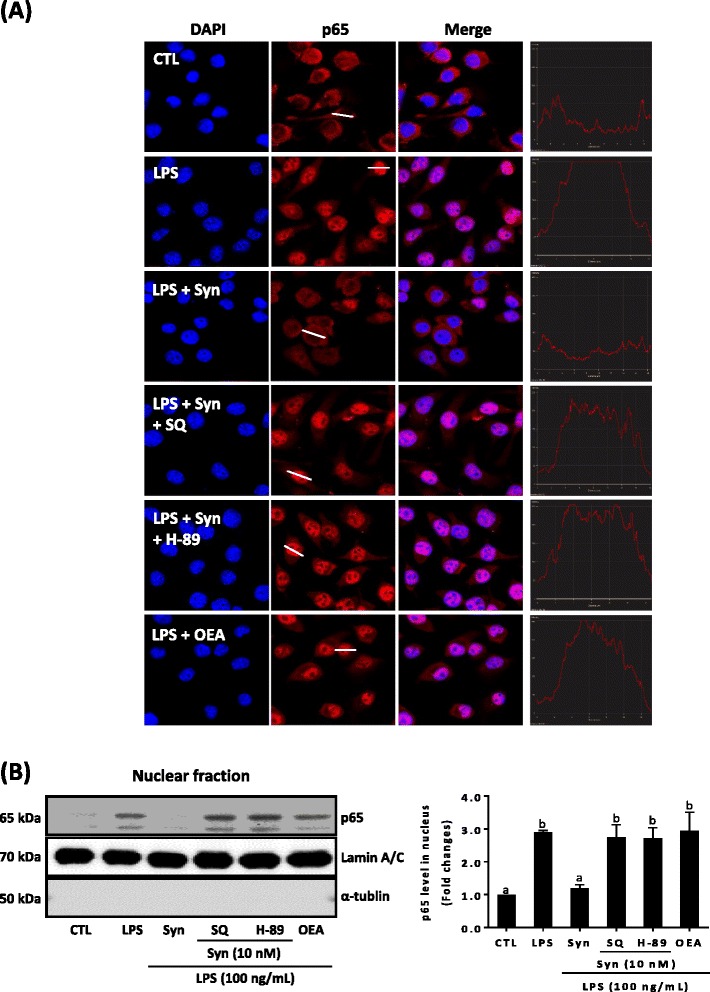
Inhibition of LPS-induced p65 translocation by synaptamide. BV2 murine microglia cells were incubated with LPS (100 ng/mL) with or without synaptamide (10 nM) and OEA (10 nM) for 30 min. Adenylyl cyclase inhibitor (SQ 22536, 10 μM) and PKA inhibitor (H-89, 10 μM) were added 30 min prior to LPS/synaptamide treatment. Translocation of p65 was determined by immunocytochemistry (*red*, Alexa Fluor 588; *blue*, DAPI) (**a**). Nuclear levels of p65 were determined by immunoblot analysis. α-Tublin and lamin A/C were used as internal controls for cytosolic and nuclear fractions (**b**). Densitometric analysis of the western blots is represented as the mean band density normalized to lamin A/C. Values are the means ± SEM (*n* = 3). Means designated with the same letter are not significantly different. In A, the fluorescence intensity profile across a transverse section of one cell indicated in the second column is shown in the fourth column

Previous studies have suggested that NF-κB is an important regulator of the TNF-α and iNOS gene expression [24, 25]. Other studies have also shown that cAMP inhibits the induction of a distinct set of genes regulated by NF-κB [22, 26, 27]. Since synaptamide induces cAMP production in microglia, we examined whether synaptamide affects p65 nuclear translocation, a downstream event of NF-κB activation (Fig. 4). Both immunostaining data (Fig. 4a) and western blot analysis after nuclear fractionation (Fig. 4b) consistently confirmed that cytosolic p65 translocates to the nucleus following LPS stimulation, and this translocation was markedly prevented by the addition of 10 nM synaptamide. Furthermore, pretreatment of BV2 cells with SQ 22536 and H-89 for 30 min abolished the synaptamide effects on LPS-induced p65 nuclear translocation, indicating that synaptamide inhibits p65 translocation through activating the cAMP/PKA signaling pathway. Thus, the suppression of LPS-induced NF-κB activation/p65 translocation through cAMP increase and PKA activation may be associated with the observed inhibitory effect of synaptamide on TNF-α and iNOS expression. A fatty acyl ethanolamide derived from oleic acid, OEA, showed no effect on p65 nuclear translocation.

### Synaptamide suppresses proinflammatory responses in LPS-challenged mice

**Fig. 5:**
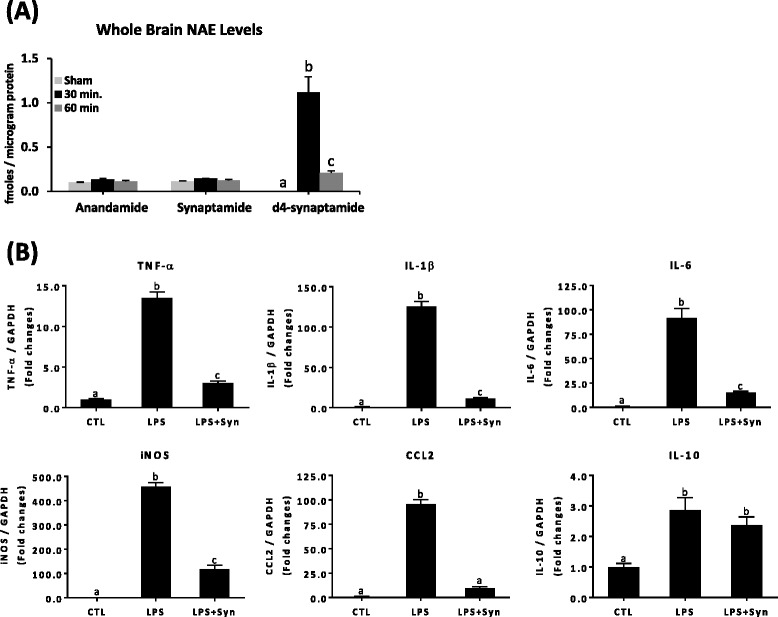
In vivo effect of synaptamide on proinflammatory responses in LPS-challenged mice. The time course of the exogenous synaptamide was determined by injecting 5 mg/kg (i.p.) d4-synaptamide into C57BL/6 mice (*n* = 3 for each time point), and brains were collected at indicated time points for LC/MS analysis (**a**). C57BL/6 mice (*n* = 3 for each group) were treated with synaptamide (5 mg/kg, i.p.) following LPS injection (1 mg/kg, i.p.), and brains were collected at 2 h to determine LPS-induced gene expression by qPCR (**b**). Values are presented as mean ± SEM from four mice. Means designated with the same letter are not significantly different

The in vivo effect of synaptamide on neuroinflammation was tested in mice after LPS-challenge (1 mg/kg, i.p.), an established model of neuroinflammation [28, 29], followed by i.p. injection of synaptamide (5 mg/kg). To confirm the synaptamide delivery to the brain, we made a parallel injection of d4-synaptamide (5 mg/kg, i.p.) and its level in the brain was evaluated at 30 min and 1 h after injection (Fig. 5a). At 30 min and 1 h after injection, d4-synaptamide was detected in the brain at 1.12 ± 0.17 and 0.21 ± 0.02 fmol/μg protein, which was approximately ten- and two-fold higher than the endogenous synaptamide level (0.11 ± 0.01 fmol/μg protein), respectively. The endogenous anandamide level (0.10 ± 0.003 fmol/μg protein) was similar to that of synaptamide. As reported earlier, LPS administration (1 mg/kg, i.p.) significantly elevated the expression of proinflammatory genes in the mouse brain (Fig. 5b). At 2 h after LPS injection, mRNA levels of TNF-α, IL-1β, IL-6, iNOS, and CCL2 increased 13.48 ± 1.73-, 125.26 ± 13.21-, 91.83 ± 13.43-, 457.11 ± 30.34-, and 95.82 ± 10.70-fold, respectively. Subsequent injection of synaptamide significantly dampened the LPS-induced increase of these proinflammatory mediators, as the LPS-induced mRNA expression of TNF-α, IL-1β, IL-6, iNOS, and CCL2 was decreased by 80.31, 90.80, 83.61, 74.46, and 90.01 %, respectively. In contrast to these proinflammatory mediators, the anti-inflammatory cytokine IL-10 that was elevated after the LPS injection was not affected by synaptamide.

### Synaptamide inhibits LPS-induced activation of microglia in the mouse brain

**Fig. 6:**
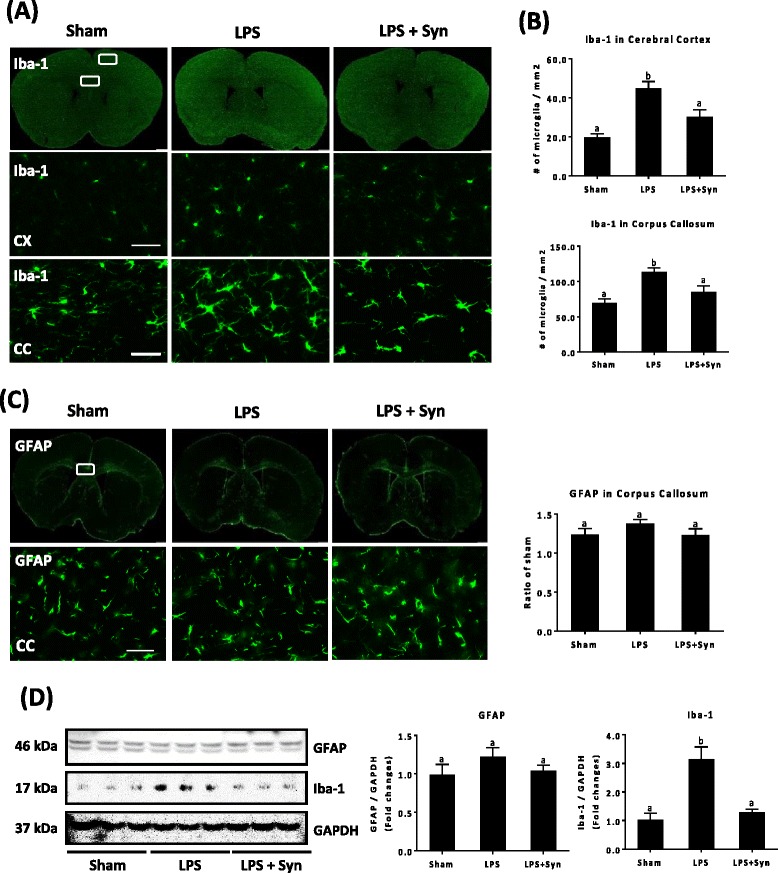
Activation of microglia suppressed by synaptamide in the LPS-challenged mouse brain. Brain sections (30-μm thick) were prepared 24 h after LPS and/or synaptamide injection, and immunostaining for Iba-1 and GFAP was performed. Iba-1 (**a**) and GFAP (**c**) images from whole sections (*top panel*) and higher magnification of the rectangular area (×20, *bottom panel*) in the cerebral cortex (CX) and corpus callosum (CC) are presented, along with quantification results of Iba-1 (**b**) and GFAP (**c**). The Iba-1 and GFAP protein levels are shown by the western blot analysis of the whole brain extract (**d**). Values are presented as mean ± SEM from four mice. Means designated with the same letter are not significantly different. Scale bar: 100 μm (**a**, **c**)

Since activated microglia and astrocytes are known to release proinflammatory mediators in the brain parenchyma [30–32], we examined whether synaptamide affects the activation of microglia and/or astrocytes. Brain sections were prepared at 24 h after LPS and synaptamide injections and immunostained with antibody against microglia and astrocyte markers Iba-1 and GFAP, respectively. The number of activated microglia that showed strong Iba-1 signal and enlarged cell body increased at 24 h after LPS injection in the cerebral cortex and corpus callosum (Fig. 6a, b). Subsequent synaptamide treatment significantly reduced Iba-1 immunoreactivity, compared to mice injected with LPS alone. In contrast to microglia, LPS at this dose did not significantly affect astrocyte activation as the GFAP immunoreactivity was not altered after LPS challenge or by synaptamide treatment (Fig. 6c). Western blot analysis also indicated that synaptamide significantly lowered the LPS-induced Iba-1 protein expression without altering the GFAP level (Fig. 6d).

### Synaptamide suppresses proinflammatory responses in LPS-challenged FAAH KO mice

**Fig. 7:**
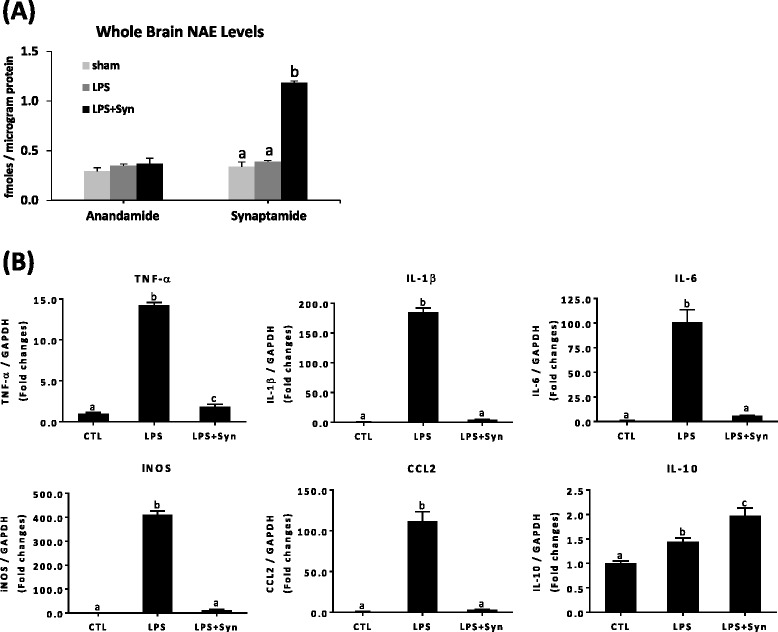
Suppression of proinflammatory responses by synaptamide in FAAH KO mice. Synaptamide levels in the FAAH KO mouse brains were quantified by mass spectrometry at 1 h after injection (i.p.) of 2 mg/kg synaptamide (**a**). The cytokine/chemokine expression in the brain was determined by qPCR at 2 h after injection of 1 mg/kg LPS followed by 2 mg/kg synaptamide (**b**). Values are presented as mean ± SEM from four mice. Means with the same letter are not significantly different

Synaptamide, as a substrate for fatty acid amide hydrolase (FAAH) [13], can be converted to DHA in vivo, and therefore, the observed anti-inflammatory effect of synaptamide may be due to its hydrolysis product DHA. To test this possibility, anti-inflammatory effects of synaptamide were examined in FAAH knock-out (KO) mice where hydrolysis of synaptamide to DHA does not occur (Fig. 7). The endogenous brain synaptamide level in FAAH KO mice (0.34 ± 0.04 fmol/μg protein, Fig. 7a) was higher compared to the level observed in the wild-type (WT) mice (0.11 ± 0.01 /μg protein, Fig. 5a). Anandamide level was also higher in FAAH KO (0.29 ± 0.04 fmol/μg protein, Fig. 7a) compared to WT brains (0.10 ± 0.003 fmol/μg protein, Fig. 5a). When synaptamide (2 mg/kg, i.p.) was injected in FAAH KO mice, brain synaptamide at 1 h after injection increased by 3.5-fold to 1.18 ± 0.02 fmol/μg protein. Upon injection of LPS (1 mg/kg, i.p.) followed by synaptamide (2 mg/kg), LPS-induced expression of TNF-α, IL-1β, IL-6, iNOS, and CCL2 was almost completely blocked (by 87.01, 97.74, 94.22, 97.03, and 97.06 %, respectively) (Fig. 7b), indicating that synaptamide suppresses the expression of proinflammatory mediators more potently in FAAH KO mice compared to WT. These results established that the inhibitory effect of synaptamide on neuroinflammation does not involve its hydrolysis to DHA. Interestingly, synaptamide treatment increased IL-10 expression in the brains of LPS-challenged FAAH KO, unlike the wild-type case.

## Discussion

In this study, we investigated potential inhibitory effects of synaptamide, a DHA metabolite, on neuroinflammation using in vitro and in vivo models of LPS-induced neuroinflammation. The main finding of this study is that synaptamide potently inhibits LPS-induced proinflammatory cytokine expression and oxidative stress in microglia by upregulating cAMP/PKA signaling and suppressing NF-κB p65 nuclear translocation. To our knowledge, this is the first demonstration of the protective role of synaptamide in LPS-induced neuroinflammation and the molecular mechanism responsible for the anti-inflammatory effects.

cAMP is an important intracellular regulator of microglia cell homeostasis and its negative perturbation through proinflammatory signaling results in microglial cell activation [33, 34]. Synaptamide was shown to induce neurogenic differentiation of NSCs [12, 14] and axon growth [12] by promoting cAMP/PKA signaling. We found that synaptamide suppresses proinflammatory cytokine expression also in a cAMP/PKA-dependent manner in LPS-treated rat and murine microglia cells. It has been reported that oxygenated forms of synaptamide block platelet-leukocyte aggregation [18]. For neuroinflammation, however, inhibiting COX, LOX, or P450 showed no influence on the synaptamide effects (Fig. 3a). Rather, inhibiting adenylyl cyclase by SQ 22536 or PKA by H-89 as well as PKA gene silencing abolished the synaptamide effects on LPS-induced TNF-α and iNOS mRNA expression in microglia cells (Fig. 3b–e), indicating that the observed anti-inflammatory effect of synaptamide was mediated through cAMP/PKA signaling. Elevation of intracellular cAMP (Fig. 3f) along with increased phosphorylation of PKA and CREB in microglia cells observed after synaptamide treatment (Fig. 3g) further supports the involvement of cAMP/PKA signaling in amelioration of LPS-induced neuroinflammation by synaptamide.

LPS activates NF-κB, an important regulator of the immune responses [35–37], through stimulation of Toll-like receptors (TLRs). NF-κB activation involves translocation of p50/p65 subunits of NF-κB from the cytoplasm to the nucleus in most cell types [38]. In microglia, LPS was shown to activate the NF-κB signaling pathway via TLR4/MyD88, which in turn promotes transcription of proinflammatory genes [39]. While LPS induces NF-κB translocation into the nucleus and its transcriptional activity [40], activation of cAMP/PKA signaling inhibits nuclear translocation of NF-κB [22, 23] and negatively modulates NF-κB transcriptional activity [22, 23, 41]. Our data indicate significant suppression of LPS-induced NF-κB p65 nuclear translocation by the cAMP-elevating metabolite synaptamide in microglia cells. The ability of synaptamide to activate cAMP/PKC was essential for this suppression as adenylyl cyclase inhibitor SQ22536, PKA inhibitor H-89, or PKA gene silencing completely blocked the observed effect of synaptamide (Fig. 4).

Activated PKA can interact with NF-κB and CREB for the regulation of gene transcription. Some studies have indicated that PKA suppresses NF-κB activity by promoting its interaction with CBP and/or p300 [42], while others have suggested that phosphorylated CREB inhibits NF-κB activation by blocking the interaction of CBP and NF-κB complex [43]. Nevertheless, overexpression of CBP and p300 failed to reverse the PKA-mediated inhibition of p65 [22], suggesting that further studies are needed to unveil the role of these proteins as well as diverse inflammation-associated genes regulated by the interaction of CREB, NF-κB, and synaptamide in future.

The anti-inflammatory effect of synaptamide was also demonstrated in vivo in the present study using a systemic LPS administration model. The levels of cytokines, nitric oxide, prostaglandins, and other proinflammatory substances in the brain increase in this model [28, 29, 44–46], shifting the CNS environment towards a proinflammatory state. Our study also showed sharp increases in the expression of TNF-α, IL-1β, IL-6, IL-10, CCL2, and iNOS mRNA after intraperitoneal injection of LPS (Figs. 5 and 7). Although how peripheral endotoxin contributes to neuroinflammation remains unclear, intercommunication between CNS and peripheral immune systems in response to endotoxin is apparent. Systemic LPS could induce brain inflammation through indirect mechanisms such as altering permeability of the blood-brain barrier (BBB), enhancing interaction between the BBB and immune cells [28, 47], or peripheral release of inflammatory substances that can cross the BBB [48, 49].

Synaptamide being a lipophilic compound clearly crossed the BBB (Figs. 5a and 7a) and produced marked suppression of LPS-induced proinflammatory cytokine/chemokine expression (Figs. 5b and 7b). At 60 min after synaptamide injection at a lower dose (2 mg/kg, i.p.), a significantly higher level of synaptamide (1.18 ± 0.02 fmol/μg protein) remained in FAAH KO brains (Fig. 7a) compared to the WT injected at 5 mg/kg, i.p. (Fig. 5a) (0.21 ± 0.02 fmol/μg protein), indicating that hydrolysis of synaptamide by FAAH actively occurred. The better inhibitory outcome after synaptamide injection observed in the FAAH KO mice was consistent with a higher synaptamide level in the KO mouse brain compared to the WT brain. These data clearly indicated that the anti-inflammatory action of synaptamide was most likely produced by synaptamide itself, not by its hydrolysis to DHA.

Previous studies have indicated some anti-inflammatory effects of AEA, OEA, and PEA at micromolar concentrations [50, 51]. However, none of these fatty acylethanolamides except synaptamide were found to be effective in suppressing proinflammatory cytokine expression at low nM concentrations (Fig. 2, Additional file 1: Figure S4). The potency and specificity of synaptamide demonstrated in our study suggest the involvement of receptor-mediated processes. Further studies will be necessary to elucidate the definitive receptor signaling that mediates the anti-inflammatory action of synaptamide.

Recently, it has been reported that it is microglia, not astrocytes, that produce IL-1β in response to TLR activation and play a pivotal role in LPS-mediated toxicity [52, 53]. In line with this report, LPS elicited microglia activation in vivo without significant induction of astrocyte activation (Fig. 6). Both in vivo (Figs. 4, 5, and 6) and in vitro results (Figs. 1, 2, 3, and 4) from the present study indicate that synaptamide is a potent regulator of LPS-induced microglial activation, suggesting microglia activation as the major target for the regulatory action of synaptamide on LPS-induced neuroinflammation. Interestingly, synaptamide exhibited stronger inhibitory effects in vivo compared to in vitro culture systems. It is possible that systemically injected synaptamide may have affected not only the microglia cells in the brain but also the peripheral immune system. Indeed, our preliminary results indicated that synaptamide suppresses LPS-induced TNF-α and IL-1β expression in the plasma at 2 and 6 h after LPS injection (Additional file 1: Figure S3). Further systematic evaluation of the synaptamide effects on peripheral immune responses will help to clarify the profound in vivo effect of synaptamide.

## Conclusions

We have demonstrated in this study that synaptamide, a DHA-derived endogenous metabolite, exerts potent anti-inflammatory effects by upregulating cAMP/PKA signaling and inhibiting NF-κB activation in microglia cells under LPS stimulation. Synaptamide-mediated inhibition of microglia activation may not only serve as a new DHA-derived neuroprotective mechanism but also provide a potential therapeutic strategy for controlling neuroinflammation and related neurodegenerative conditions.

## Additional file

Additional file 1:
**Figure S1.** Purity of microglia cells in culture assessed by immunofluorescence detection of Iba-1- (microglia marker, red), GFAP- (astrocyte marker, green), or O4-positive cells (oligodendrocyte marker, green). The purity of the Iba-1-positive microglia was >90 %. **Figure S2.** Dose- and time-dependent effect of LPS on TNF-α expression in BV2 murine microglia cells. Cells were incubated with LPS (ng/mL) at indicated concentration for 30 min, 1 h, and 4 h. Data are presented as mean ± SEM (*n* = 3). The statistical significance was evaluated by Student’s *t* test compared with control (CTL). **Figure S3.** Effect of synaptamide on the plasma proinflammatory cytokines production in LPS-challenged mice. C57BL/6 mice (*n* = 3 for each group) were treated with LPS (1 mg/kg, i.p.) followed by synaptamide (5 mg/kg, i.p.), and blood was collected at 2 and 6 h. Cytokine levels in the plasma were determine by ELISA. Data are presented as mean ± SEM. The statistical significance was evaluated by Student’s *t* test compared with the LPS treatment. **Figure S4.** Suppression of LPS-induced IL-1β, IL-6, and CCL2 expression specifically by synaptamide. BV2 cells were treated with LPS (100 ng/mL), and 10 nM fatty acyl ethanolamides were added immediately after the LPS exposure. Levels of IL-1β, IL-6, and CCL2 mRNA were determined by qPCR after 1 h incubation. The data represent the mean ± SEM of three independent experiments. The statistical significance was evaluated by Student’s *t* test compared with the LPS treatment. (PPT 464 kb)

## Abbreviations

AEA: Arachidonoyl ethanolamine
ANOVA: Analysis of variance
BBB: Blood-brain barrier
cAMP: Cyclic adenosine monophosphate
CCL2: Chemokine (C-C motif) ligand 2
CNS: Central nervous system
COX: Cyclooxygenase
CREB: cAMP response element binding protein
DAPI: 4′,6-Diamidino-2-phenylindole
DHA: Docosahexaenoic acid
ELISA: Enzyme-linked immunosorbent assay
FAAH: Fatty acid amide hydrolase
GAPDH: Glyceraldehyde-3-phosphate
GFAP: Glial fibrillary acidic protein
Iba-1: Ionized calcium binding adaptor molecule 1
IL: Interleukin
iNOS: Inducible nitric oxide synthase
KO: Knock out
LOX: Lipoxygenase
LPS: Lipopolysaccharide
LSD: Least significant difference
NF-κB: Nuclear factor kappa light chain enhancer of activated B cells
NSC: Neural stem cell
OEA: *N*-Oleoylethanolamine
P450: Cytochrome P450
PEA: Palmitoyl ethanolamine
PKA: Protein kinase A
qPCR: Quantitative polymerase chain reaction
SD: Standard deviation
SEM: Standard error of mean
TLR: Toll-like receptor
TNF: Tumor necrosis factor
