# Supplementary material for: N-Docosahexaenoylethanolamine ameliorates LPS-induced neuroinflammation via cAMP/PKA-dependent signaling
Source: J Neuroinflammation. 2016 Nov 4;13:284. doi: 10.1186/s12974-016-0751-z (PMC5096293; doi:10.1186/s12974-016-0751-z)
Supplement: Additional file 1: — Figure S1. Purity of microglia cells in culture assessed by immunofluorescence detection of Iba-1- (microglia marker, red), GFAP- (astrocyte marker, green), or O4-positive cells (oligodendrocyte marker, green). The purity of the Iba-1-positive microglia was >90 %. Figure S2. Dose- and time-dependent effect of LPS on TNF-α expression in BV2 murine microglia cells. Cells were incubated with LPS (ng/mL) at indicated concentration for 30 min, 1 h, and 4 h. Data are presented as mean ± SEM (n = 3). The statistical significance was evaluated by Student’s t test compared with control (CTL). Figure S3. Effect of synaptamide on the plasma proinflammatory cytokines production in LPS-challenged mice. C57BL/6 mice (n = 3 for each group) were treated with LPS (1 mg/kg, i.p.) followed by synaptamide (5 mg/kg, i.p.), and blood was collected at 2 and 6 h. Cytokine levels in the plasma were determine by ELISA. Data are presented as mean ± SEM. The statistical significance was evaluated by Student’s t test compared with the LPS treatment. Figure S4. Suppression of LPS-induced IL-1β, IL-6, and CCL2 expression specifically by synaptamide. BV2 cells were treated with LPS (100 ng/mL), and 10 nM fatty acyl ethanolamides were added immediately after the LPS exposure. Levels of IL-1β, IL-6, and CCL2 mRNA were determined by qPCR after 1 h incubation. The data represent the mean ± SEM of three independent experiments. The statistical significance was evaluated by Student’s t test compared with the LPS treatment. (PPT 464 kb) [file 12974_2016_751_MOESM1_ESM.ppt]

## Slide 1
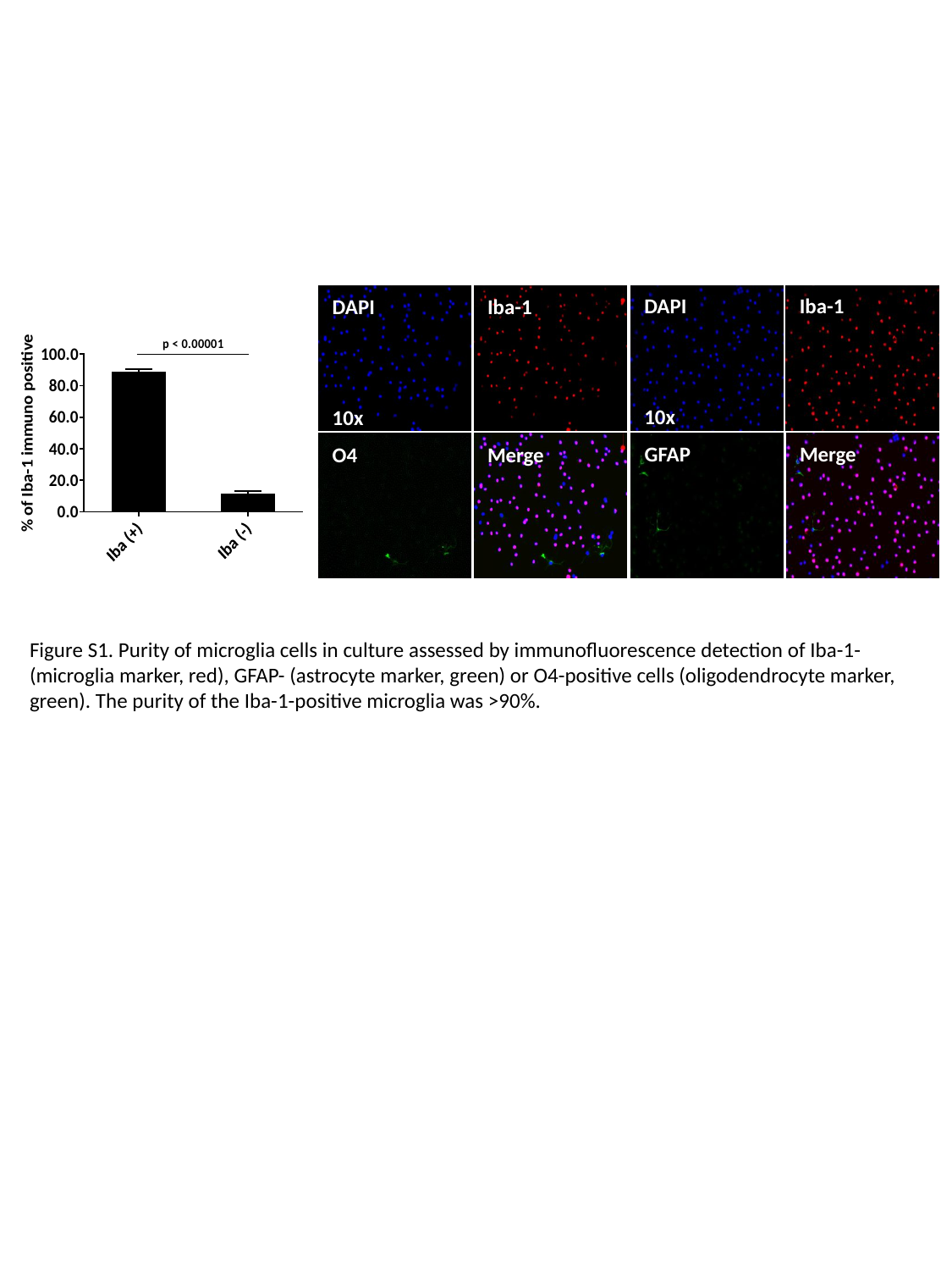

DAPI
Iba-1
DAPI
Iba-1
10x
10x
GFAP
Merge
O4
Merge
Figure S1. Purity of microglia cells in culture assessed by immunofluorescence detection of Iba-1- (microglia marker, red), GFAP- (astrocyte marker, green) or O4-positive cells (oligodendrocyte marker, green). The purity of the Iba-1-positive microglia was >90%.

## Slide 2
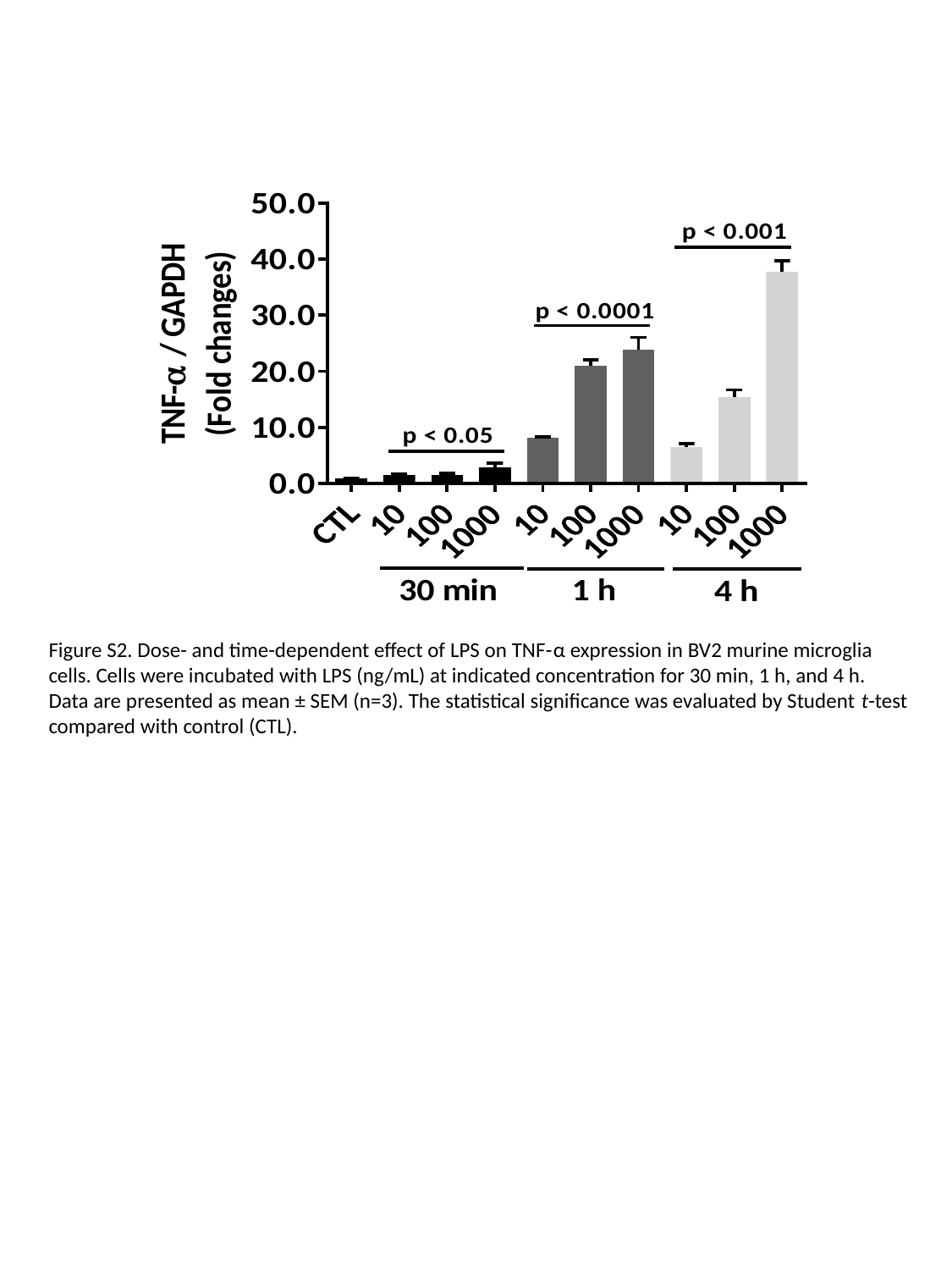

Figure S2. Dose- and time-dependent effect of LPS on TNF-α expression in BV2 murine microglia cells. Cells were incubated with LPS (ng/mL) at indicated concentration for 30 min, 1 h, and 4 h. Data are presented as mean ± SEM (n=3). The statistical significance was evaluated by Student t-test compared with control (CTL).

## Slide 3
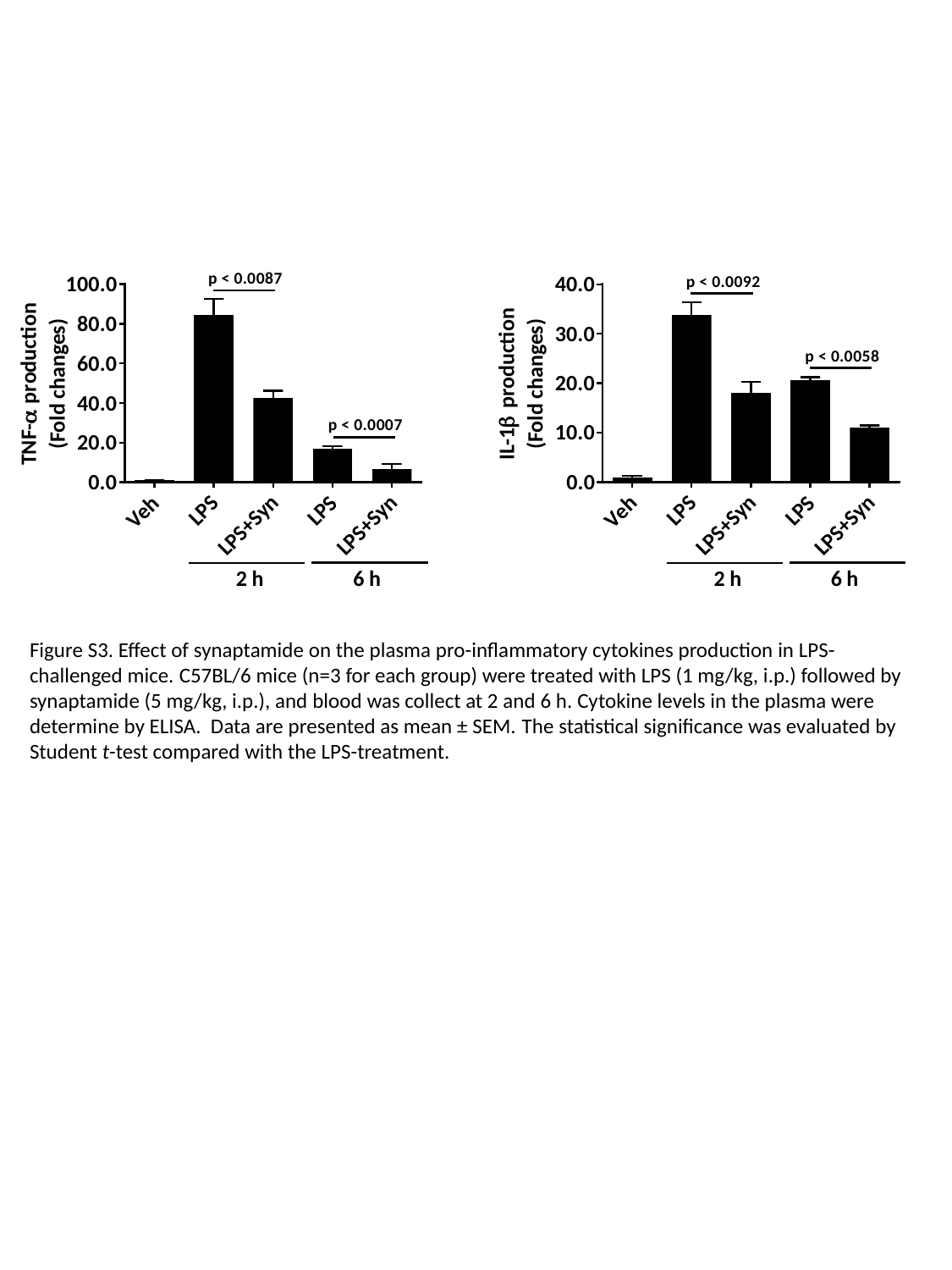

Figure S3. Effect of synaptamide on the plasma pro-inflammatory cytokines production in LPS-challenged mice. C57BL/6 mice (n=3 for each group) were treated with LPS (1 mg/kg, i.p.) followed by synaptamide (5 mg/kg, i.p.), and blood was collect at 2 and 6 h. Cytokine levels in the plasma were determine by ELISA. Data are presented as mean ± SEM. The statistical significance was evaluated by Student t-test compared with the LPS-treatment.

## Slide 4
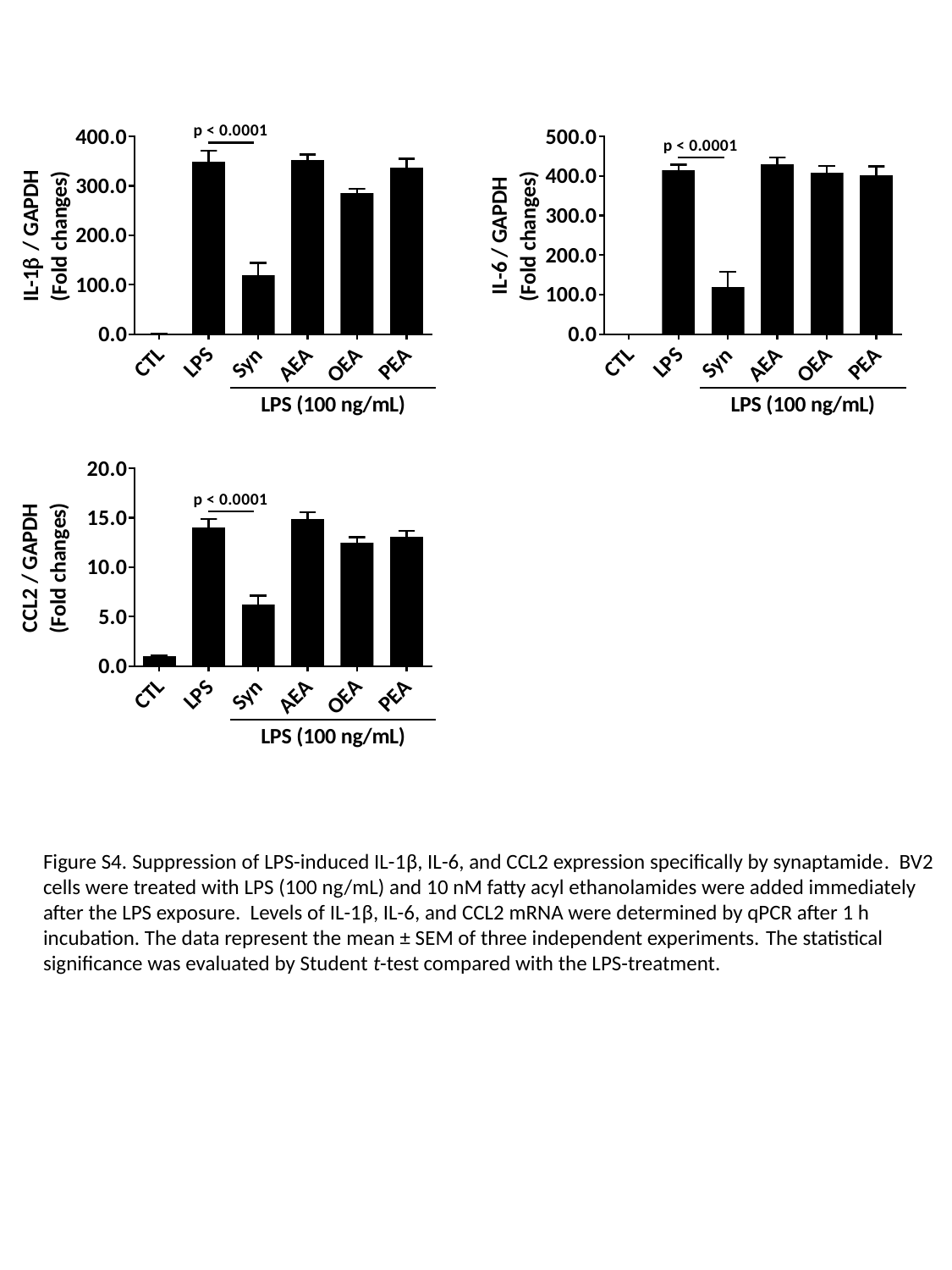

Figure S4. Suppression of LPS-induced IL-1β, IL-6, and CCL2 expression specifically by synaptamide. BV2 cells were treated with LPS (100 ng/mL) and 10 nM fatty acyl ethanolamides were added immediately after the LPS exposure. Levels of IL-1β, IL-6, and CCL2 mRNA were determined by qPCR after 1 h incubation. The data represent the mean ± SEM of three independent experiments. The statistical significance was evaluated by Student t-test compared with the LPS-treatment.
